# Estradiol Reverses Ovariectomy-Induced Disruption of Hypothalamic Gene Expression and Behavior via Modulation of Gonadotropin Releasing Hormone and Calcium Signaling Pathways

**DOI:** 10.3390/ani15101467

**Published:** 2025-05-19

**Authors:** Asim Muhammad, Mubashir Muhammad, Xiaohuan Chao, Chunlei Zhang, Jiahao Chen, Huan Yang, Shuhan Liu, Yuan Ding, Ziming Wang, Hongwei Bi, Wen Guo, Junhong Fan, Bo Zhou

**Affiliations:** College of Animal Science and Technology, Nanjing Agricultural University, Nanjing 210095, China; 2022105147@stu.njau.edu.cn (A.M.); mubashir@stu.njau.edu.cn (M.M.); 2021205020@stu.njau.edu.cn (X.C.); 2020105039@stu.njau.edu.cn (C.Z.); 2021105039@stu.njau.edu.cn (J.C.); 2021105038@stu.njau.edu.cn (H.Y.); 2022105002@stu.njau.edu.cn (S.L.); 2023105045@stu.njau.edu.cn (Y.D.); 2023805171@stu.njau.edu.cn (Z.W.); 2023805170@stu.njau.edu.cn (H.B.); 2024805170@stu.njau.edu.cn (W.G.); 2024105048@stu.njau.edu.cn (J.F.)

**Keywords:** ovariectomy, estradiol intervention, hypothalamic transcriptomics, GnRH signaling pathway, calcium signaling pathway, reproductive aging

## Abstract

Estrogen plays a crucial role in regulating reproductive and neuroendocrine functions, yet the molecular mechanisms underlying its effects on the hypothalamus remain incompletely understood. This study investigates the transcriptional and behavioral changes induced by ovariectomy (OVX) and estradiol (E2) supplementation in female C57BL/6J mice. RNA sequencing was performed to identify differentially expressed genes (DEGs) across control (CK), E2, OVX, and OVX+E2 groups, followed by functional enrichment and pathway analyses. Behavioral assessments, including open field, Y-maze, and elevated plus maze tests, were conducted to evaluate anxiety-like and cognitive behaviors. Results revealed significant alterations in GnRH signaling, neurotransmission, and inflammatory pathways, with key genes such as *Elk1*, *Prkcb*, and *Camk2a* differentially expressed in response to estrogen modulation. OVX-induced neuroendocrine disruptions were partially reversed by E2 treatment, as evidenced by transcriptomic and behavioral outcomes. Pearson correlation analysis further linked gene expression patterns with phenotypic traits, providing insights into estrogen’s regulatory mechanisms in the hypothalamus. These findings enhance our understanding of estrogen-mediated neuroendocrine regulation and may have implications for hormone replacement therapies in postmenopausal disorders.

## Simple Summary

This research analyzes ovariectomy-induced ovariectomy and estradiol supplementation effects on hypothalamic genomic patterns and female behavioral responses in mice. RNA sequencing analysis reveals substantial regulatory changes in the GnRH pathway and calcium signaling pathway. Ovariectomy procedures triggered anxiety-like behavior, weight gain, and food consumption rise, yet estrogen therapy provided partial remedy for these effects. Behavioral tests with correlation analysis revealed three important genes (*Elk1*, *Prkcb*, and *Camk2a*) that act as regulators of neuroendocrine control mechanisms. This study finds data linking estrogen effects on hypothalamic functionality and shows potential therapeutic potential for postmenopausal medical conditions.

## 1. Introduction

Reproduction is an important aspect for species survival as it ensures gene transmission to the next generation. It has been established that neurobiological fluctuations in the central nervous system (CNS) and peripheral nervous system (PNS) are associated with reproductive behaviors [1]. During the premenstrual, postpartum, and menopausal stages of a woman’s reproductive life, numerous hormonal changes take place in the brain and plasma that affect physiological and emotional functioning.

In rodents, the hypothalamic–pituitary–gonadal (HPG) axis controls reproduction [2]. The hypothalamus is a neuroendocrine center positioned upstream of the HPG axis [3]. It can initiate puberty by controlling endocrine, circadian, and gonadal development [4]. The hypothalamus releases gonadotropin-releasing hormones (GnRHs), which stimulate the pituitary gland to control the release of luteinizing hormone (LH) and follicle-stimulating hormones (FSHs), which act on the ovaries and control the production of gonadal steroid hormones. The release of GnRHs is carefully regulated through feedback mechanism involving sex hormones, such as testosterone in men and estradiol in women. Therefore, the hypothalamus is crucial for the sexual development of mammals [5]. The neuro-regulation of GnRH is multifaceted, involving neurotransmitters from various hypothalamic regions [6]. KNDy neurons, located in different parts of the hypothalamus, play a crucial role in the GnRH pulse generator and are linked to the early stages of reproductive decline in women and female rats, including enhanced follicular atresia in middle-aged women [7]. In summary, regulating GnRH pulses along with the activity of upstream KNDy and GABA neurons may drive reproductive aging and offer a potential target for intervention.

17β-Estradiol (E2), the principal endogenous estrogen, is primarily synthesized in the ovaries and is essential for the development of the female reproductive system, maintenance of secondary sexual characteristics, regulation of the menstrual cycle, and support of pregnancy [8]. Acting through estrogen receptors (ERα and ERβ), ligand-activated transcription factors expressed in diverse tissues, including the brain, E2 signaling mediates critical adaptive processes across physiological systems, with particularly pronounced effects on reproductive neuroendocrinology. In neural tissues, these receptors modulate mood, sexual behavior, and cognitive functions, underscoring their role in integrating hormonal and behavioral homeostasis [9]. Preclinical studies in rodents demonstrate that ovariectomy-induced estrogen deprivation elevates inflammatory markers in brain regions such as the frontal cortex and hippocampus [10], mimicking pathological pathways observed in menopausal women. Clinically, postmenopausal estrogen deficiency is associated with increased risks of metabolic dysfunction, hypertension, and neuropsychiatric disorders, highlighting the far-reaching consequences of hormonal imbalance on systemic health [11].

Hormone replacement therapy (HRT), involving estrogen and progesterone, is a primary intervention for managing menopausal symptoms [12]. However, the balance of benefits and risks associated with HRT is influenced by variables such as age, menopausal symptom severity, and individual risk profiles [13,14], underscoring the need for developing alternative interventions that promote menopausal health without adverse effects. Ovariectomy in preclinical models, including mice and pigs, induces anxiety- and depression-like behaviors alongside disruptions in reproductive cyclicity, processes regulated by hypothalamic GnRH signaling [15]. Here, we hypothesize that estradiol and ovariectomy exert bidirectional impacts on the HPG axis, a key regulatory hub for reproductive physiology in mice. To test this, we investigated how ovariectomy, with or without estradiol supplementation, alters HPG axis function, linking hormonal manipulations to molecular and behavioral outcomes.

A previous study has reported significant hypothalamic transcriptomic alterations in ovariectomized versus intact mice [16]. However, RNA sequencing investigations into hypothalamic gene expression dynamics under varying estrogen concentrations, specifically following ovariectomy and subsequent hormone replacement, remain limited. This study aims to characterize the effects of ovariectomy on hypothalamic gene expression profiles and behavioral phenotypes, while also evaluating the potential restorative effects of E2 supplementation. Ovariectomy induces profound molecular and behavioral changes mediated by neuroendocrine pathways, including GnRH signaling, neurotransmission, and inflammatory processes. Estradiol supplementation is hypothesized to mitigate these alterations by re-establishing estrogen-dependent gene regulation and signaling networks that govern reproductive physiology and behavioral outputs. By investigating differentially expressed genes and enriched biological pathways, this study seeks to delineate the molecular underpinnings of menopausal symptoms and inform the development of targeted therapies for hormone deficiency-related disorders.

## 2. Materials and Methods

### 2.1. Animals and Treatments

Forty female C57BL/6J mice (8 weeks old, 22–24 g) were purchased from Jiangsu Extractive Pharmaceutical and Biotechnology Co., Ltd. (Nanjing, China) and housed at the Experimental Center of Nanjing Agricultural University under pathogen-free conditions. The mice were maintained at 23 °C and 45% humidity on a 12-h light/dark cycle with ad libitum access to sterile food and water. Mice randomly selected for ovariectomy were anesthetized via intraperitoneal injection of tribromoethanol (0.2 mL; Nanjing AIBI Bio-Technology Co., Ltd., Nanjing, China). The lower back region was clipped and disinfected sequentially with povidone–iodine and ethanol swabs. A small incision was made through the abdominal musculature to expose the uterine horn; following ligation of the oviduct, both ovaries were excised. The muscular layer was sutured with absorbable thread, and the skin was closed with surgical staples before the mice were transferred to individually ventilated recovery cages. Postoperative monitoring included daily assessment for complications, such as ≥10% body weight loss, signs of infection, or incomplete ovarian removal. After a one-week acclimation period, animals were assigned to four experimental groups (*n* = 10 per group): control (CK), estradiol-treated (E2), ovariectomized (OVX), and ovariectomized with estradiol treatment (OVX+E2).

E2 was purchased from Med Chem Express (Nanjing, China) and dissolved in sesame oil. Mice in the E2 and OVX+E2 groups received daily intraperitoneal injections of E2 (0.2 mg E2/kg body weight dissolved in sesame oil) for 13 days. This dosage was based on reference [17] and validated in preliminary trials to ensure physiological relevance, while the OVX and CK groups received an equivalent volume of sesame oil as a vehicle control. This E2 dosage was approximately two to three times higher than the normal circulating levels in mice under standard maintenance conditions [18]. Drug administration was conducted at 11:00 a.m. daily. Feed intake was recorded daily, and body weight was measured every three days to monitor physiological responses and ensure the well-being of the animals throughout the study.

### 2.2. Vaginal Smear

Daily vaginal smears were collected before assigning the mice to the experimental groups. This study included only females with three regular, uninterrupted cycles (four to five days). Only cycling mice were included to accurately identify the ovarian cycle phase, as required by the experimental design. To standardize the vaginal smear procedure, the same technique was applied to each experimental group. Vaginal smears were collected daily at 10:00 a.m. by gently inserting the rounded tip of an eye swab into the vaginal opening, moving it in and out, and then transferring the fluid onto a microscope slide. The ovarian cycle phase was immediately assessed using an optical microscope (40× magnification). Smears were classified as proestrus, estrus, meta estrus, or diestrus based on the approximate proportions of leukocytes, nucleated epithelial cells, and cornified epithelial cells [19].

### 2.3. Serum Hormone Concentration Test

Following 13 days of treatment, blood samples (500 μL) were collected via orbital venipuncture at 10:00 a.m. to ensure temporal consistency and minimize circadian rhythm-related variability. Serum concentrations of E2 were quantified using a commercial ELISA kit (Lot no.: G20241118GG; Shanghai Lengton Bioscience Co., Ltd., Shanghai, China), with all procedures performed according to the manufacturer’s instructions. Similarly, GnRH levels were assayed using a validated ELISA kit (Lot no.: G20240829CQ; Shanghai Lengton Bioscience Co., Ltd., Shanghai, China), adhering strictly to the provided protocol. The minimum detection limit for both assays was 1.0 pg/mL. Throughout the experiment, animal handling and care were conducted in compliance with guidelines approved by the Institutional Animal Care and Use Committee of Nanjing Agriculture University (SYXK SU 2022-0031), prioritizing welfare and minimizing stress to experimental subjects.

### 2.4. Behavioral Tests

**Figure 1:**
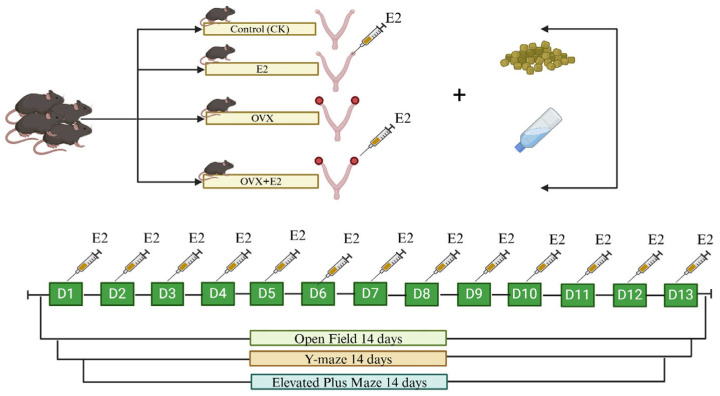
Animals and treatments.

To assess the impact of ovariectomy and E2 treatments on anxiety, depression, and memory impairment, mice were tested in the open field (5 min), Y-maze (8 min), and elevated plus maze (5 min) (Figure 1). A gap of approximately 30 min was provided between each test, as previously described [20] (Table S1, Table S2, Table S3, Supplementary File S1).

### 2.5. Open Field Test (OFT)

OFT was conducted according to the method described in the existing literature [21]. A 10 × 10 cm^2^ central area was defined in the field box (40 × 40 × 40 cm^3^). The experiment allowed each mouse to freely explore the open field for five continuous minutes, starting from the center. An above-arena Canon EOS Rebel SL3/EOS 250D high-resolution camera recorded all sessions was sourced from Canon Inc., headquartered in Tokyo, Japan. A software tracking program known as ANY-maze, developed by (Stoelting Europe, Ireland, Dublin). The version of ANY-maze used during our experiments was Version 7.43, released in 2021, recorded both distances traveled, and durations spent in the central area [22]. On the test day, the following factors were assessed: (a) time spent in the center zone, (b) total number of entries into the center zone, and (c) frequency of wall contacts. The anxiety index was calculated according to Cohen et al. [23].

### 2.6. Y-Maze

Referring to previous research [24], a single arm of the apparatus converged towards a central angle while the remaining two arms remained parallel to each other. Each arm had dimensions of 40 × 25 × 6 cm. The natural exploratory behavior of mice leads them to investigate areas they have never entered previously. The mice received 8 min of open exploration time within the maze. The experimental activity was captured through a Canon EOS Rebel SL3/EOS 250D high-resolution camera system (Canon Inc., headquartered in Tokyo, Japan). The mouse reached an arm when its entire body crossed the entry barrier with its four legs. An alternate arm entry occurred when mice moved between three distinct arms, one after another. The maximum possible number of arms was determined through a subtraction of two from the complete arm entries count. A percentage alternate behavior score was obtained by dividing the total alternate arm entries by the maximum number of arm entries and multiplying that total by 100%.

### 2.7. Elevated Plus Maze (EPM)

The elevated plus maze (EPM) functions as a behavioral screening method for detecting anxiety behaviors in rodents [25]. ANY-maze tracking software (Stoelting Europe, Ireland, Dublin) was used; the apparatus was white and consisted of runways (5 cm wide × 35 cm long) with perpendicular wall-to-wall compartments (5 × 5 cm). The experiment used two pairs of runways that differed in boundary conditions: one pair had tall walls (called closed arms; height of 15 cm), and the other pair had no walls (open arms). The maze was elevated 40 cm above the floor. The Canon EOS Rebel SL3/EOS 250D digital camera (Canon Inc., headquartered in Tokyo, Japan) maintained a position above the apparatus during the 5-min test period to record mouse movements. Two independent observers analyzed recorded behavior until reaching a 95% threshold. The following parameters were assessed: (a) time spent in the open arms, (b) total number of arm entries, and (c) number of entries into the open arms. The anxiety index was calculated as described by Cohen et al. [26]. After each mouse completed their trial, the EPM was cleaned with 70% ethanol to eliminate the odor cues.

### 2.8. Sample Collection and RNA Sequencing

After two weeks of estrogen administration, all mice were euthanized under tribromoethanol anesthesia. A surgical procedure was performed to immediately excise and store the hypothalamus, liver, ovary, and uterus. These tissues were rapidly frozen in liquid nitrogen and stored in 1.5-mL microtubes. A total of 40 samples were collected from each experimental group. The GnRH ELISA test was performed according to the kit’s instructions (Lot no.: G20240829CQ; Shanghai Lengton Bioscience Co., Ltd., Shanghai, China).

Total RNA was extracted from the hypothalamus tissue using TRIzol reagent (Invitrogen, Carlsbad, CA, USA). RNA concentration and purity were measured using the Qubit RNA Assay Kit on a Qubit 2.0 Fluorometer (Life Technologies, Carlsbad, CA, USA) and a NanoPhotometer^®^ Spectrophotometer (IMPLEN, Westlake Village, CA, USA). RNA-seq was performed using six samples from each group of mice (n = 10). The mice were weighed two weeks after the procedure began and anesthetized deeply with tribromoethanol. After decapitation, the heads were placed on ice, and a craniotomy was performed to extract the hypothalamus, which was preserved in liquid nitrogen. The samples were sequenced by Beijing Bamak Biotech Co., Ltd. (Beijing, China) [27].

RNA-seq libraries were constructed from hypothalamic tissue samples of C57BL/6J mice. Clean reads were derived from these samples and assessed for quality using Trimmomatic [28], which included calculations for Q20, Q30, and GC content metrics. The HISAT2 v2 aligner was then used during (24 September 2024) to map the clean reads to the mouse reference genome (GRC-M39, http://asia.ensembl.org/mus_musculus/Info/Index) [29].

### 2.9. Identification of Differentially Expressed Genes

The number of reads in each sample was quantified using the “FeatureCounts” tool. Differential expression between the four experimental groups was analyzed using the “DESeq2” program version 1.30.1 [30]. The threshold for differential expression was set to an adjusted *p*-value < 0.05 and |log2 (fold change)| > 1 for the following comparisons: CK vs. E2, CK vs. OVX, CK vs. OVX+E2, E2 vs. OVX, E2 vs. OVX+E2, and OVX vs. OVX+E2. This approach was used to identify differentially expressed genes in hypothalamic tissues.

### 2.10. Analysis of the Important Pathways and the Functions of Hypothalamus Tissue

Fold change values for all genes across the four groups were calculated using the “DESeq2” package and sorted in descending order. These values were used as input for gene set enrichment analysis (GSEA) using the “DAVID Functional Annotation Bioinformatics Microarray Analysis” tool, which included six comparisons of hypothalamic tissues from the four groups. Pathways and Gene Ontology (GO) terms that were significantly enriched (*p*-value < 0.05) were identified. Additionally, gene set variation analysis (GSVA) was conducted using key pathways as input [31]. For each pathway in each sample, enrichment scores (ESs) were calculated. The pathways showing significant enrichment in at least one group were examined and visualized using “chiplot” (https://www.chiplot.online/), (accessed on 12 October 2024).

### 2.11. RT-qPCR Verification

Total cDNA was synthesized using a reverse transcriptase kit from CWBIO (Nanjing, China). RT-qPCR was performed on a QuantStudio 10 using SuperStar Universal SYBR Green Master Mix, also obtained from (CWBIO, Beijing, China). PCR reactions were conducted in triplicate using primers designed with the Primer Premier 5 program (Table S4) as mentioned in (Supplementary File S1). Relative gene expression was calculated using the 2^−ΔΔCt^ method, with β-actin as the reference gene. Data were analyzed using IBM SPSS Statistics version 22.0 software (IBM Corp., Armonk, NY, USA).

### 2.12. Statistical Analysis

The statistical analyses were conducted using SPSS 22.0 software (IBM Corp., Armonk, NY, USA). One-way analysis of variance (ANOVA) was used to assess differences among groups in body weight, feed intake, tissue gonadotropin-releasing hormone (GnRH) levels, serum hormone levels, and behavioral parameters. Pearson correlation analysis was performed to evaluate associations between phenotypic data and gene expression levels. To identify significant gene–gene interactions, a multivariate analysis was conducted using SAS software version 9.4 (https://welcome.oda.sas.com/login) (accessed on 20 January 2025). For qRT-PCR analysis, statistical comparisons were performed using SPSS 22.0, with data expressed as mean ± standard error (SEM). Group comparisons were conducted using the least significant difference (LSD) test, following verification of normality (Shapiro–Wilk test) and homogeneity of variances (Levene’s test). Additionally, Duncan’s multiple range test was applied for post hoc comparisons. Independent sample t-tests were also performed for pairwise comparisons. Behavioral data from the open field, Y-maze, and elevated plus maze tests were analyzed using ANY-maze software. Figures were generated using GraphPad Prism 5 (GraphPad Software, San Diego, CA, USA). Post hoc analyses using Duncan’s least significant difference (LSD) test assigned statistical group labels, where groups sharing at least one common letter exhibit no statistically significant differences, whereas groups with entirely distinct letters indicate significant differences.

## 3. Results

### 3.1. Development of a Model Menopause and Vaginal Smear

**Figure 2:**
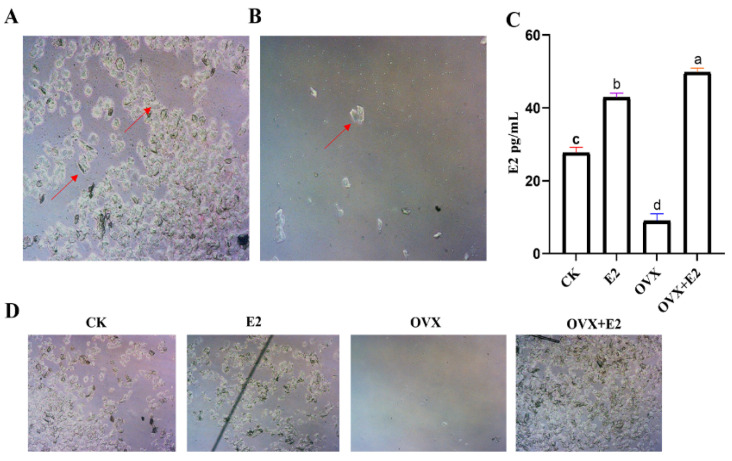
Illustrates the successful outcome of an ovariectomy in mice. (**A**,**B**) The results of vaginal exfoliated cells obtained from mice in the OVX+E2 and OVX groups exhibit a disruption of the estrous cycle (as indicated by the presence of nucleated and non-nucleated epithelial cells in the OVX+E2 and OVX groups as shown by red arrows in the figure). (**C**) The serum estrogen levels of the mice in the four experimental groups (i.e., CK, E2, OVX, and OVX+E2). Two weeks following ovariectomy, mice in the OVX group exhibited significantly lower blood estrogen levels than those in the CK, E2, OVX, and OVX+E2 groups. (**D**) The daily observation of estrus in conjunction with CK, E2, OVX, and OVX+E2. Different lowercase letters in the figure indicate statistically significant differences (*p* < 0.05).

Vaginal cytology analyses revealed distinct cyclic patterns in exfoliated vaginal cells among the CK, E2, and OVX+E2 groups when compared to the OVX group, as depicted in Figure 2A,B. Cells in each experimental group exhibited abundant cytoplasm but displayed distinct morphological features: CK and E2 groups showed organized cellular structures consistent with normal estrous cycling, whereas OVX group cells frequently exhibited atypical morphology, including large leukocyte clusters, isolated nuclei, and nuclear fragments. Conversely, Figure 2C demonstrates that the OVX+E2 group had significantly lower serum E2 levels than both the CK and E2 groups, reflecting partial hormonal restoration after E2 supplementation in ovariectomized animals. Daily monitoring of estrous stages revealed significant between-group differences in cyclicity (Figure 2D, *p* < 0.05), with the OVX group showing disrupted estrus patterns characterized by prolonged diestrus and reduced estrus duration, while CK and E2 groups maintained regular cyclicity.

### 3.2. Consequence of Ovariectomy and 17β-Estradiol Replacement on Body Weight and Food Intake

**Figure 3:**
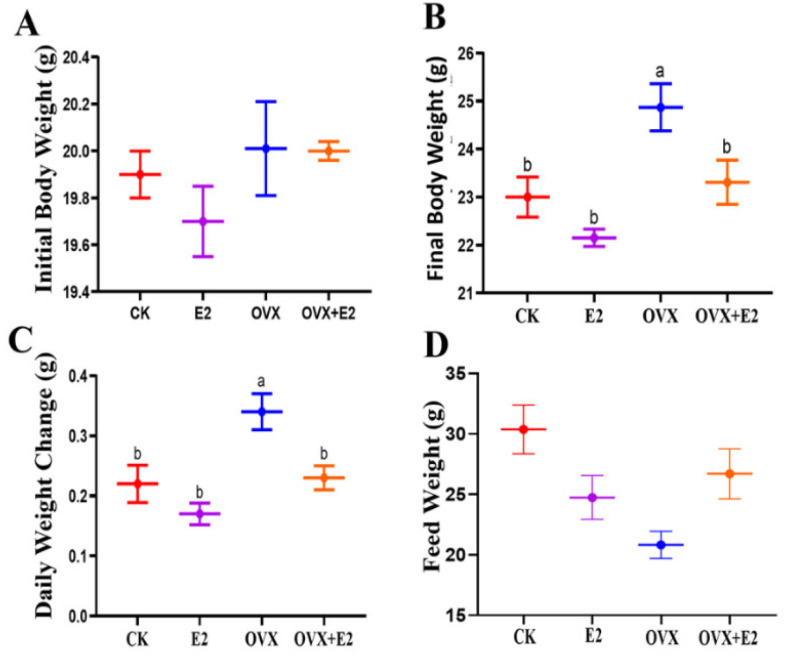
Changes in body weight and food intake across the experimental groups. These changes are shown as follows: initial body weight (**A**), final body weight (**B**), daily weight gain (**C**), and food intake (**D**) in the control (CK), estradiol (E2), ovariectomized (OVX), and OVX+E2 mice. Data are presented as mean ± SEM, with n = 10 for each group. Significant differences (*p* < 0.05) between the CK and treated (E2, OVX, OVX+E2) groups were determined using the LSD and Duncan tests. Different lowercase letters in the figure indicate statistically significant differences (*p* < 0.05).

At the study’s onset (8 weeks of age), no significant weight differences were observed among the treatment groups, as documented in Figure 3A. By 10 weeks post-procedure, OVX animals exhibited a substantial increase in body weight compared to the CK, E2, and OVX+E2 groups. This weight gain trend persisted through week 14, as illustrated in Figure 3B. Concomitant with their accelerated weight gain, the OVX group displayed a 10% increase in food intake relative to other groups. Between weeks 12 and 14, coinciding with the initiation of 17β-estradiol treatment in relevant groups, weight gains in the CK, E2, and OVX+E2 groups remained significantly lower than in the OVX group (Figure 3C). Group-specific differences in weight trajectories, including OVX, CK, E2, and OVX+E2, remained consistent throughout the study period. As shown in Figure 3D, variations in feed consumption across experimental groups indicated a correlation between dietary behavior and body weight dynamics during the investigation of ovariectomy combined with hormonal therapy.

### 3.3. Ovariectomy and 17β-Estradiol Replacement and Their Interactive Impact on Mice Anxiety

To evaluate the endocrine changes induced by ovariectomy, estradiol supplementation was administered to assess anxiety-like behavior in mice. The experimental protocol involved estradiol supplementation combined with exposure to the Y-maze (Y), as well as preliminary testing using the elevated plus maze (EPM) and open field test (OFT) paradigms. These assessments were conducted throughout the experimental period, from the 1st day to the 13th day.

### 3.4. Result of the Open Field Test (OFT)

**Figure 4:**
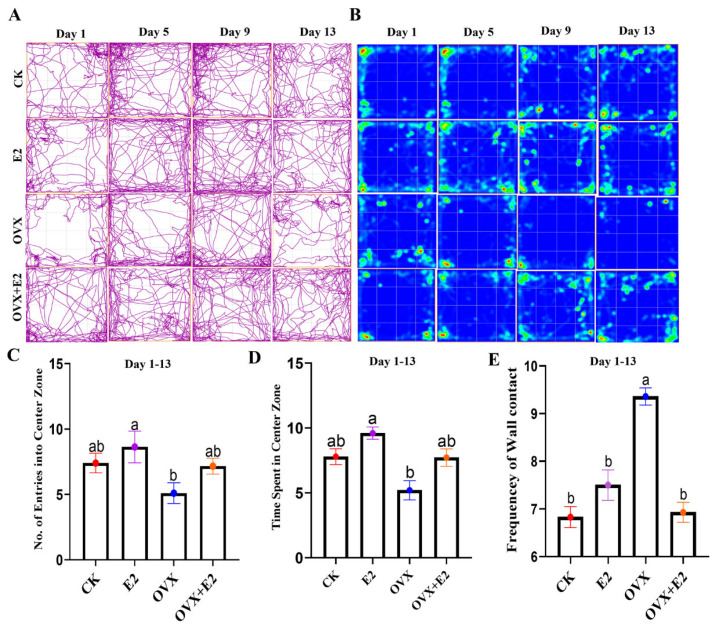
The open field (OF) test at 1 to 13 days. (**A**) Representative 2D tracking image, (**B**) heatmap, (**C**) number of entries into the center zone, (**D**) time spent in the center zone, and (**E**) frequency of wall contact. Data are presented for the control (CK), estradiol-treated (E2), OVX, and estradiol-treated OVX (OVX+E2) groups. Significant differences were determined by LSD and Duncan tests, different lowercase letters in the figure indicate statistically significant differences (*p* < 0.05).

A representative 2D tracking image and heatmap (Figure 4A,B) are shown to visualize the movement patterns. Statistical analysis revealed a significant main effect of estradiol (E2) administration and ovariectomy on the total number of entries into the center zone of the mice in the CK, E2, and E2-treated OVX mice, as compared to the OVX group (*p* < 0.05). The results showed that E2-treated mice entered the central zone more frequently than the OVX mice (Figure 4C). Furthermore, mice treated with E2 spent more time in the center zone compared to OVX mice (*p* < 0.01; Figure 4D). In contrast, a significant difference in the frequency of wall contact was observed between the CK, E2, and OVX+E2 groups, as compared to the OVX group (*p* < 0.01; Figure 4E).

### 3.5. Result of the Y-Maze

**Figure 5:**
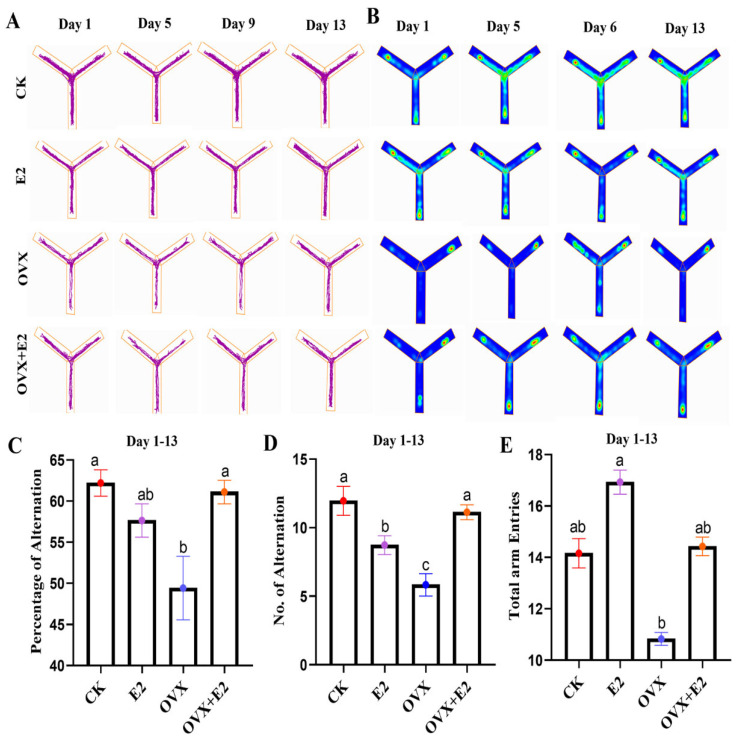
Y-maze tests at 1 to 13 days. (**A**) Representative 2D tracking image, (**B**) heatmap, (**C**) percentage of alternation, (**D**) number of alternations, and (**E**) total arm entries. Data are presented for the control (CK), estradiol-treated (E2), OVX, and estradiol-treated OVX (OVX+E2) groups. Significant differences were determined by LSD and Duncan tests, different lowercase letters in the figure indicate statistically significant differences (*p* < 0.05).

Representative 2D tracking images and heatmaps are shown in Figure 5A,B. The percentage of alternations differed significantly among the experimental groups (*p* < 0.01), with the OVX+E2 group exhibiting a lower alternation percentage (*p* < 0.01) compared to the CK, OVX, and E2 groups (Figure 5C). Analysis of variance revealed significant differences in alternation frequency after six days of treatment (*p* = 0.03 and *p* = 0.003; Figure 5D). Furthermore, the total number of arm entries was significantly higher in the CK group and lower in the OVX+E2 group compared to the OVX and E2 groups (*p* < 0.001 and *p* < 0.01, respectively; Figure 5E).

### 3.6. Result of the Elevated Plus Maze (EPM)

**Figure 6:**
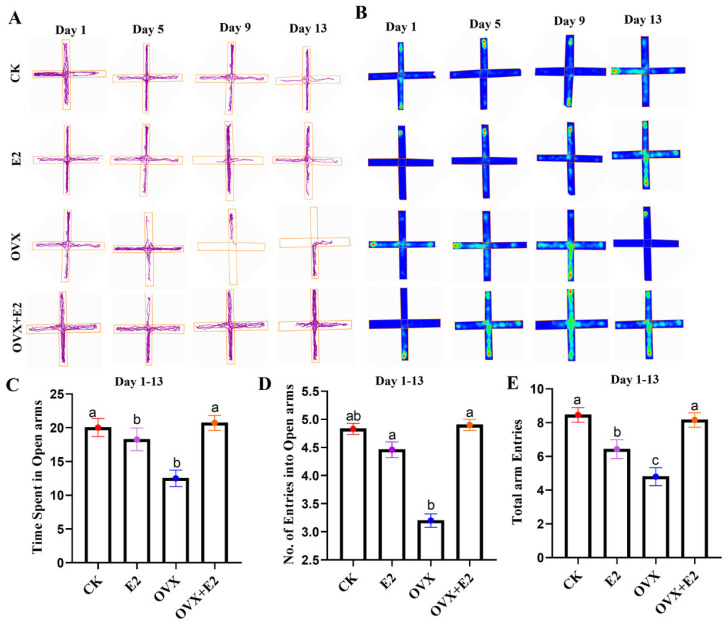
Elevated plus maze (EPM) tests at 1 to 13 days. (**A**) Representative 2D tracking image, (**B**) heatmap, (**C**) time spent on the open arm, (**D**) number of entries into the open arm, and (**E**) total arm entries. Data are presented for the control (CK), estradiol-treated (E2), OVX, and estradiol-treated OVX (OVX+E2) groups. Significant differences were determined by LSD and Duncan tests, different lowercase letters in the figure indicate statistically significant differences (*p* < 0.05).

Representative 2D tracking images and a heatmap are provided in (Figure 6A,B). A statistically significant difference (*p* < 0.001) was observed in the amount of time spent on the open arms among the various groups. Compared to the OVX group, the CK group and all treated groups except for the OVX group that received estradiol treatment demonstrated a reduced time spent on the open arms (*p* < 0.001) (Figure 6C). Additionally, a significant difference (*p* < 0.001) was detected in the number of open-arm entries across the groups. The OVX+E2 group showed a reduced number of open-arm entries compared to the OVX group (*p* < 0.05) (Figure 6D). Furthermore, a significant difference (*p* = 0.05) was observed in the overall number of arm entries. The OVX+E2 group exhibited a significant decrease in the total number of arm entries compared to the OVX, E2, and CK groups (*p* < 0.05; Figure 6E).

### 3.7. Implication of Ovariectomy and 17β-Estradiol Substitution on GnRH of the Hypothalamus Tissue of Mice

**Figure 7:**
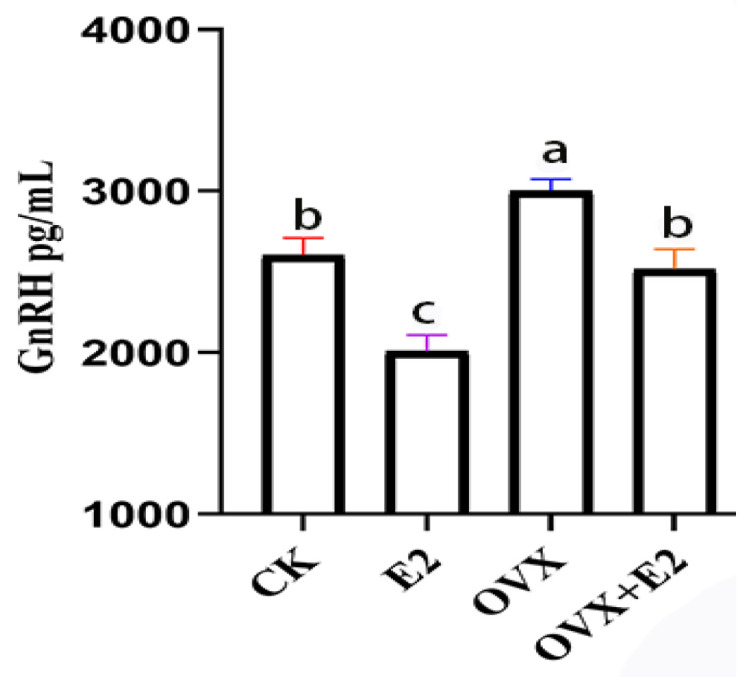
The impact of ovariectomy and 17β-estradiol supplementation on GnRH levels in mouse hypothalamic tissue. The OVX group exhibited the highest GnRH levels, while E2 treatment resulted in the lowest. Different lowercase letters in the figure indicate statistically significant differences (*p* < 0.05).

In hypothalamic tissue, the GnRH concentration in the OVX group exhibited a statistically significant increase compared to the CK, E2, and OVX+E2 groups (*p* < 0.01). Conversely, the GnRH concentration in the E2 group was significantly lower than in both the CK and OVX+E2 groups (*p* < 0.05) (Figure 7). No significant differences were observed between the CK and OVX+E2 groups, indicating that these two experimental groups were comparable.

### 3.8. Effect on Gene Expression Profiles of Mouse Hypothalamic Tissues Following Ovariectomy and 17β-Estradiol Replacement

**Figure 8:**
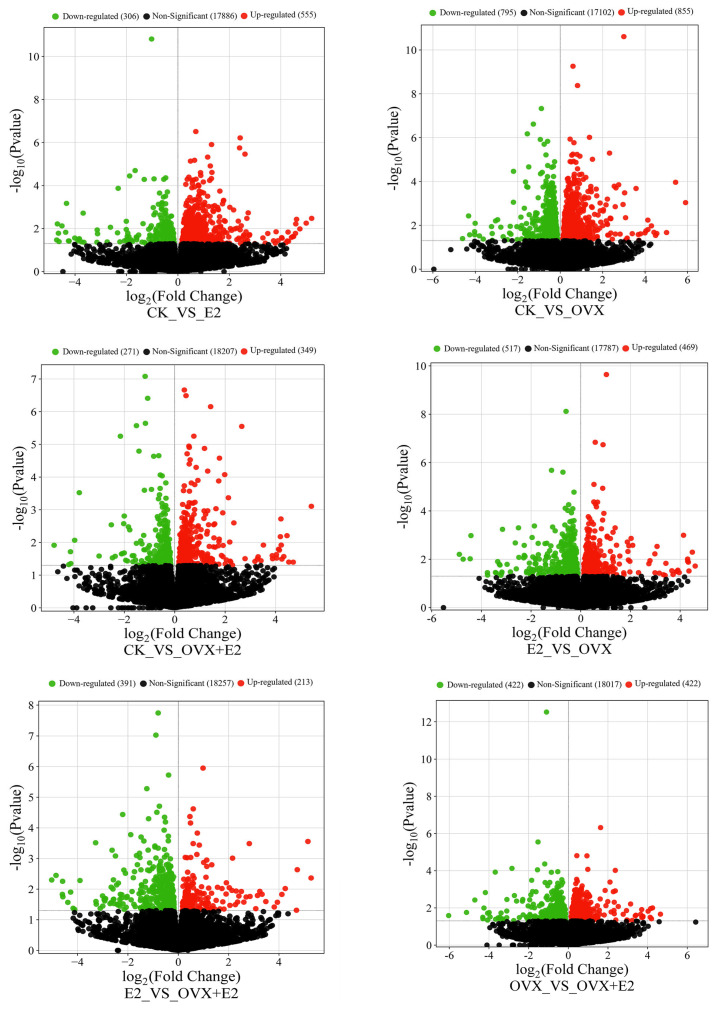
Volcano plot of differentially expressed genes (DEGs) across experimental groups. In the diagram, red indicates upregulation of DEGs and green indicates downregulation. The x-axis represents the log2-fold change, and the y-axis represents the significance value following a -log10 transformation. The comparison groups include CK_vs_E2, CK_vs_OVX, CK_vs_OVX+E2, E2_vs_OVX, E2_vs_OVX+E2, and OVX_vs_OVX+E2.

The differential gene expression (DEG) analysis was performed to evaluate the changes in gene expression among four different groups, resulting in a total of six comparative analyses. Post-treatment with E2, the differentially expressed genes (DEGs) demonstrated statistically significant alterations in their expression levels, with a notable number being upregulated. This upregulation was visually represented through volcano plots for each of the six comparisons conducted, as shown in Figure 8.

A total of 861 DEGs were identified in the comparison between the CK and E2 groups (555 upregulated and 306 downregulated); among these, key DEGs *Camk2a*, *Ptk2b*, *Calml4*, and *Elk1* were upregulated and were associated with learning and memory, cell cycle regulation, apoptosis, cell adhesion, cell migration, cell division, cytoskeletal structures, and integrated functions of differentiation and stress responses. While one gene, *Mapk1*, was downregulated and was allied with controlling signal transmission and gene regulation. Estradiol replacement in the ovariectomized mice affected 1650 transcripts (855 upregulated and 795 downregulated) in the comparison between CK and OVX groups, e.g., *Elk1* and *Camk2a* are involved in transcription regulation and calcium signaling, respectively. A total of 620 DEGs were identified between the CK and OVX+E2 groups (349 upregulated and 271 downregulated); among these, five key DEGs, *Camk2a*, *Ptk2b*, *Prkcd*, *Cacna1c*, and *Elk1*, were upregulated and were related to cell cycle regulation, apoptosis, cell adhesion, cell migration, cell division, cytoskeletal structures, and cellular calcium ion entry, which leads to muscle contraction and hormone release as well as neuronal signaling activities. A similar analysis revealed 986 DEGs between the E2 vs OVX groups (349 upregulated and 271 downregulated), and among these key DEGs, two genes, *Prkcb*, *Elk1* were upregulated and two genes, *Calml4* and *Mapk1*, were downregulated, and these genes were linked with cell division and influence cytoskeletal structures and cellular shape, signal transduction mechanisms, differentiation, and stress responses. A total of 604 DEGs were identified in the E2_vs_OVX+E2 comparison (391 upregulated and 213 downregulated in OVX+E2); among these, three key DEGs, *Calml4*, *Prkcb*, and *Mapk1*, are involved in signaling pathways that determine cell division and influence cytoskeletal structures and cellular shape.

**Figure 9:**
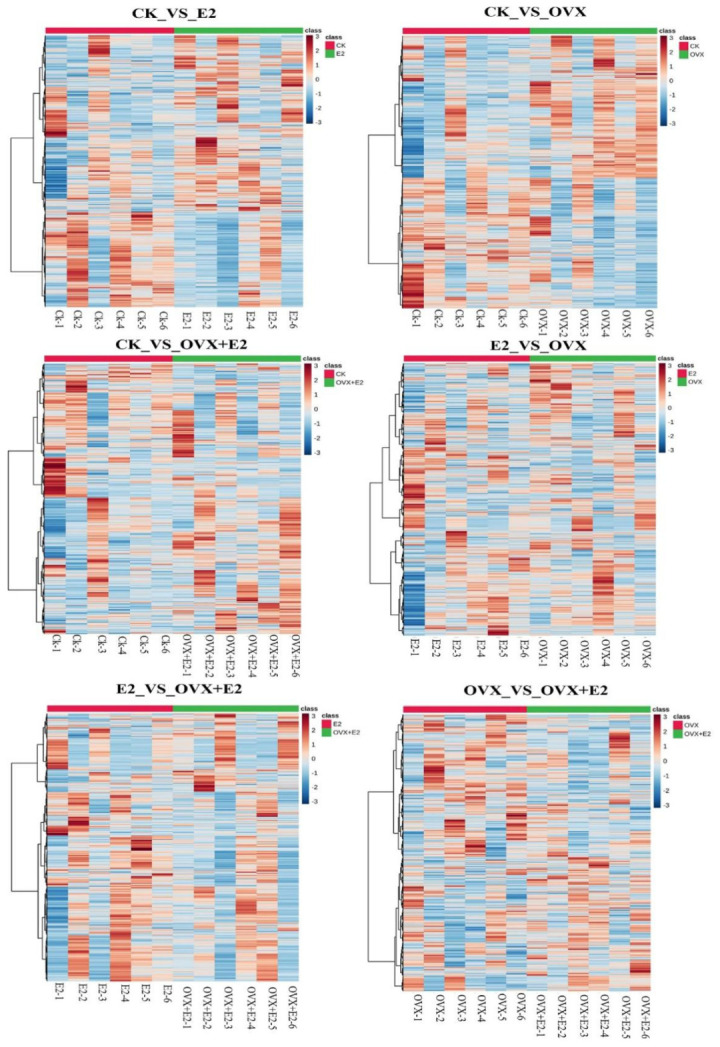
Heatmaps analyzing differential gene expression in mouse models across various treatment groups: control (CK), estradiol-treated (E2), ovariectomized (OVX), and ovariectomized with estradiol (OVX+E2). Each heatmap reveals distinct transcriptional profiles between groups, with color intensity indicating expression levels. Clustering demonstrates the impact of ovariectomy and E2 treatment on gene regulation, suggesting E2’s potential to mitigate ovariectomy-induced changes.

Finally, 844 DEGs were detected in the OVX_vs_OVX+E2 comparison 422 upregulated and 422 downregulated in OVX+E2 (Figure 9), e.g., upregulated Prkcd and downregulated *Camk2a* are involved in core synaptic plasticity agents, apoptosis, and stress response regulation. The predominant expression pattern of these DEGs showed an inverse relationship between the conditions following ovariectomy, indicating that their expression levels were counter-regulated by the administration of 17β-estradiol. The hypothalamus was significantly impacted by both ovariectomy and 17β-estradiol treatment.

Gene Ontology (GO) enrichment analysis using DAVID identified key functional pathways associated with hypothalamic responses to ovariectomy and estrogen replacement therapy across six experimental comparisons. The analysis revealed distinct molecular signatures in biological processes (BPs), cellular components (CCs), and molecular functions (MFs). Cognitive processes were prominently enriched in estrogen-treated groups: CK_vs_E2 showed significant enrichment in cognition (FDR = 0.000532), learning and memory (FDR = 0.00136), and behavior control, alongside synaptic remodeling features such as asymmetric synapses (FDR = 0.000157) and postsynaptic density (FDR = 4.98 × 10^−9^). Similar cognitive enrichment was observed in CK_vs_OVX+E2 (learning/memory, FDR = 0.000532) and E2_vs_OVX (cognition, FDR = 4.98 × 10^−9^), reinforcing estrogen’s role in maintaining cognitive function.

Ovariectomy-induced changes in CK_vs_OVX included learning and memory deficits (FDR = 0.000532), impaired behavior regulation (FDR = 0.000532), and altered glutamate receptor binding (FDR = 0.0000915), suggesting synaptic dysfunction. These deficits were partially reversed by estrogen replacement in CK_vs_OVX+E2, which also showed enrichment in reproductive-related BP terms such as placenta development (FDR = 4.98 × 10^−9^) and steroid hormone receptor activity (FDR = 4.98 × 10^−9^).

**Figure 10:**
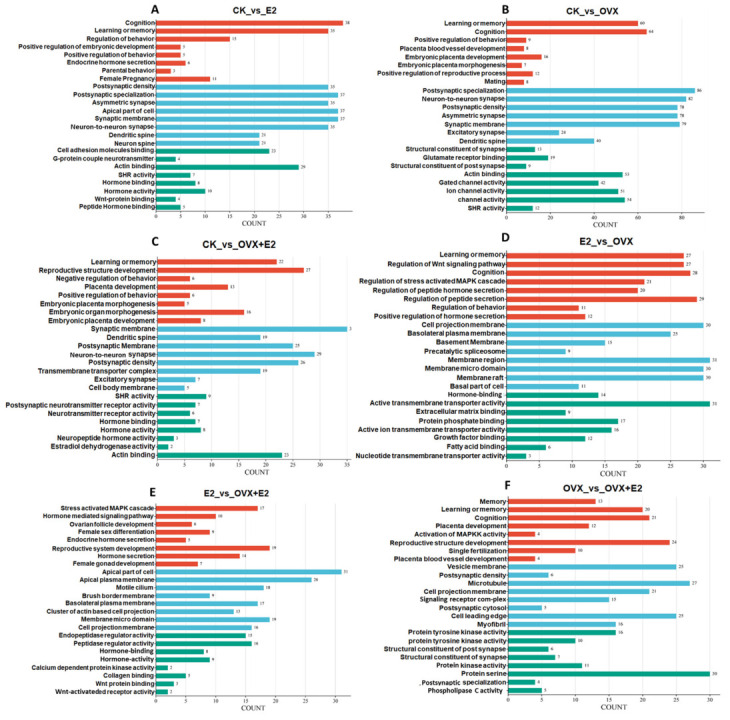
An illustration of the GO enrichment analysis for the comparison groups. (**A**) CK_vs_E2, (**B**) CK_vs_OVX, (**C**) CK_vs_OVX+E2, (**D**) E2_vs_OVX, (**E**) E2_vs_OVX+E2, and (**F**) OVX_vs_OVX+E2, after ovariectomy and 17β-estradiol treatments. The analysis highlights molecular activities, cellular components, and biological processes. The treatments tested were CK (control), E2 (0.2 mg/kg dissolved in sesame oil), OVX (ovariectomized), and OVX+E2 (0.2 mg/kg dissolved in sesame oil).

Comparisons between estrogen-treated groups highlighted dynamic membrane remodeling: E2_vs_OVX+E2 was enriched in apical plasma membrane components (FDR = 0.0000915) and motile cilia (FDR = 0.000157), while OVX_vs_OVX+E2 showed enrichment in vesicle membranes (FDR = 4.98 × 10^−9^) and protein tyrosine kinase activity (FDR = 4.98 × 10^−9^). Hormone binding emerged as a shared MF feature across estrogen-responsive groups, appearing in CK_vs_OVX+E2 (FDR = 0.000157), E2_vs_OVX+E2 (FDR = 0.000157), and E2_vs_OVX (FDR = 4.98 × 10^−9^). Collectively, these findings (Figure 10A–F) demonstrate estrogen’s dual role in preserving cognitive synaptic plasticity and regulating reproductive signaling pathways in the hypothalamus, with estrogen replacement partially mitigating ovariectomy-induced alterations.

### 3.9. KEGG Enrichment Analysis of DEGs

**Figure 11:**
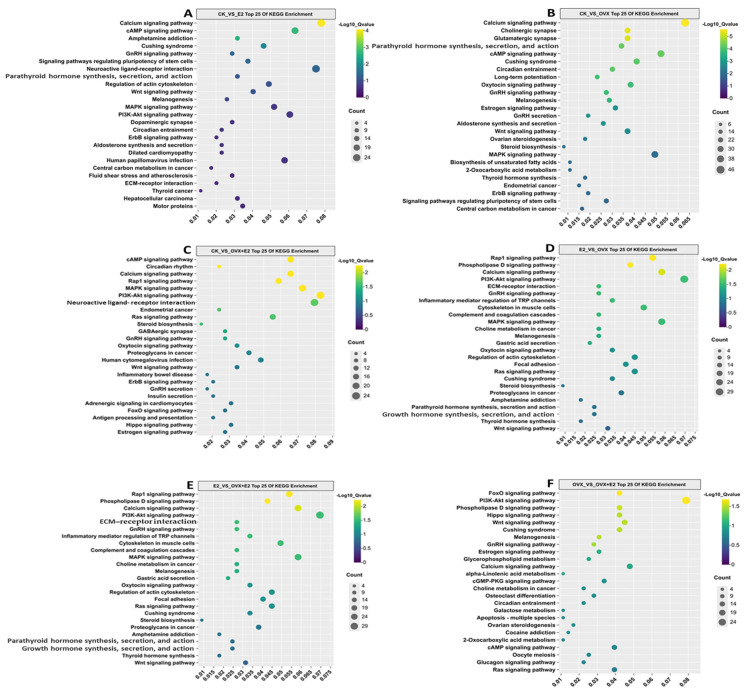
Top 25 enriched KEGG pathways for the following comparisons. (**A**) CK_vs_E2, (**B**) CK_vs_OVX, (**C**) CK_vs_OVX+E2, (**D**) E2_vs_OVX, (**E**) E2_vs_OVX+E2, and (**F**) OVX_vs_OVX+E2, following ovariectomy and 17β-estradiol treatments. The KEGG pathway is represented on the axis, with the abscissa showing the ratio of DEGs annotated to each KEGG pathway relative to the total number of DEGs. The size of the dots reflects the number of DEGs annotated to each pathway. The treatments tested were: CK (control), E2 (0.2 mg/kg dissolved in sesame oil), OVX (ovariectomized), and OVX+E2 (0.2 mg/kg dissolved in sesame oil).

KEGG enrichment analysis of differentially expressed genes (DEGs) in hypothalamic tissue samples from OVX and E2-treated mice revealed significant involvement of these genes in several key signaling pathways, including calcium signaling, GnRH signaling, MAPK signaling, oxytocin signaling, and Wnt signaling, across different treatment groups (Figure 11).

In the CK_vs_E2 comparison group, the calcium, GnRH, and MAPK signaling pathways showed the highest density of DEGs, although the neuroactive ligand–receptor interaction pathway exhibited a notably higher enrichment ratio within the same group. In the CK_vs_OVX comparison, pathways such as calcium signaling, GnRH signaling, GnRH secretion, oxytocin signaling, and cAMP signaling were significantly enriched.

In contrast, the CK_vs_OVX+E2 group showed marked enrichment in cAMP signaling, MAPK signaling, GnRH signaling, neuroactive ligand–receptor interaction, and steroid biosynthesis pathways. For the E2_vs_OVX comparison, pathways related to calcium signaling, GnRH signaling, PI3K-AKT signaling, and RAP1 were significantly enriched. Similarly, in the E2_vs_OVX+E2 group, MAPK signaling, GnRH signaling, PI3K-AKT signaling, and calcium signaling were the most significantly enriched pathways.

Finally, in the OVX_vs_OVX+E2 comparison, significant enrichment was observed in PI3K-AKT signaling, GnRH signaling, Wnt signaling, and calcium signaling pathways. A total of 1000 genes were found to be involved in these key pathways across all comparison groups (CK_vs_E2, CK_vs_OVX, CK_vs_OVX+E2, E2_vs_OVX, E2_vs_OVX+E2, and OVX_vs_OVX+E2).

### 3.10. Impact of E2 and Ovariectomy on the GnRH Signaling Pathway in Mice Hypothalamus: Implications for Reproductive Function

**Figure 12:**
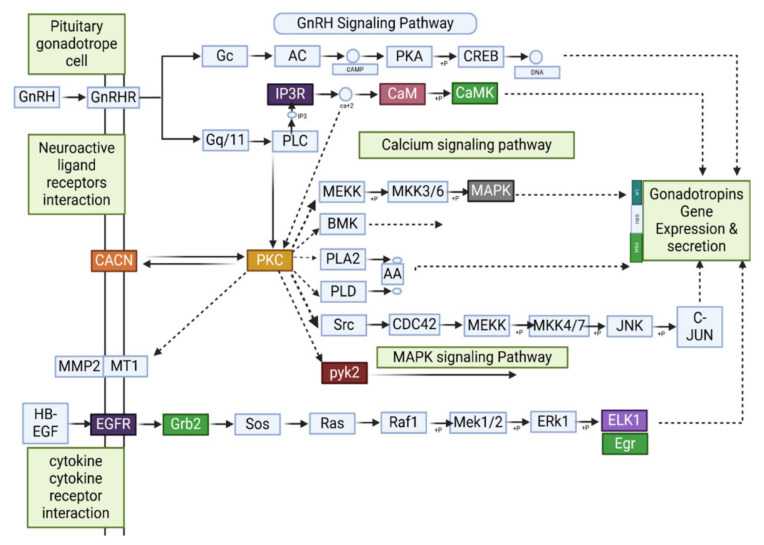
The GnRH signaling pathway involves multiple molecular interactions regulating gonadotropin gene expression and secretion. GnRH (Gonadotropin-releasing hormone) binds to GnRHR, activating intracellular cascades via *IP3R* (Inositol 1,4,5-Trisphosphate Receptor), which mediates calcium release, and *CaM* (Calmodulin), a calcium-binding messenger that activates *CaMK* (Calmodulin-Dependent Kinase), influencing gene transcription. *CACN* (Calcium Channel) facilitates calcium influx, crucial for cellular responses. *PKC* (Protein Kinase C) and *PYK2* (Proline-rich Tyrosine Kinase 2) integrate multiple signaling inputs to modulate downstream kinases. The *EGFR* (Epidermal Growth Factor Receptor) pathway, via *Grb2* (Growth Factor Receptor-Bound Protein 2), activates Ras-MAPK signaling, leading to transcription factor activation, including *ELK1* (ETS Like-1 Protein) and *Egr* (Early Growth Response Protein), which regulate target gene expression essential for reproductive function.

An investigation into the effects of ovariectomy and E2 treatment on transcriptional regulation of the GnRH signaling pathway identified the findings presented in Figure 12 through DEGs analysis. Across six intergroup comparisons involving four experimental groups, this study detected 70 DEGs.

In the CK_vs_E2 comparison, nine DEGs were implicated in the GnRH signaling pathway, with three (*Elk1*, *Ptk2b*, and *Mmp14*) upregulated and six (*Egr1*, *Fshb*, *Cga*, *Mapk12*, *Calml4*, and *Camk2a*) downregulated. In CK_vs_OVX, 16 DEGs were identified, including *Elk1*, *Ptk2b*, *Src*, *Adcy2*, *Adcy5*, *Adcy9*, *Camk2a*, *Egfr*, *Gna11*, *Gnaq*, *Mmp2*, *Mapk1*, *Nras*, *Pla2g4a*, *Plcb3*, *Plcb4*, *Pld2*, and *Prkacb*. Notably, *Elk1*, *Ptk2b*, *Src*, *Adcy2*, *Adcy5*, *Adcy9*, *Camk2a*, *Egfr*, *Gna11*, *Gnaq*, *Mapk1*, *Plcb3*, *Plcb4*, *Pld2*, and *Prkacb* were upregulated, while *Mmp2*, *Nras*, and *Pla2g4a* were downregulated.

In CK_vs_OVX+E2, five genes (*Elk1*, *Ptk2b*, *Camk2a*, *Cacna1c*, and *Prkcd*) were upregulated, whereas three (*Egr1*, *Map2k3*, and *Nras*) were downregulated. The E2_vs_OVX comparison revealed seven upregulated genes (*Elk1*, *Adcy2*, *Camk2b*, *Egr1*, *Egfr*, *Gnaq*, and *Prkcb*) and five downregulated genes (*Calml4*, *Mmp2*, *Map2k3*, *Pla2g4f*, and *Plcb2*). In E2_vs_OVX+E2, three genes (*Prkcb*, *Cga*, and *Map2k3*) were upregulated, while four (*Calml4*, *Fshb*, *Mmp14*, and *Map2k3*) were downregulated. Finally, in OVX_vs_OVX+E2, three genes (*Camk2b*, *Fshb*, and *Prkcd*) were upregulated, whereas seven (*Adcy1*, *Adcy2*, *Camk2a*, *Egfr*, *Itpr1*, *Plcb3*, and *Pld2*) were downregulated.

The experimental approach involving ovariectomy and E2 treatment induces significant alterations in the GnRH signaling pathway, concurrently modulating brain neuronal function and reproductive physiology. This investigation identified key genes within this pathway, each contributing distinct mechanistic roles. *Elk1*, a pivotal transcription factor, mediates immediate-early gene expression to modulate GnRH secretion through the MAPK/ERK signaling cascade; *Camk2a* regulates calcium-dependent neuronal excitability, influencing synaptic plasticity critical for GnRH neuronal activity; *Ptk2b* participates in integrin-mediated cell adhesion and signaling, impacting HPG axis connectivity; *Prkcd* is associated with apoptotic pathways, potentially affecting the survival of GnRH-producing neurons; *Cacna1c* encodes a voltage-gated calcium channel subunit, shaping electrical firing patterns in hypothalamic neurons; *Prkcb* modulates protein kinase C-dependent cellular processes, including differentiation and inflammatory responses in the hypothalamus; *Mapk1* (ERK2) functions within the MAPK/ERK pathway to regulate cell proliferation, stress responses, and gene transcription relevant to GnRH neuron homeostasis; and *Gna11* contributes to G-protein-coupled receptor signaling, integrating E2-mediated feedback into GnRH secretory dynamics. These findings demonstrate that GnRH signaling relies on a complex genetic architecture, with each gene orchestrating distinct molecular events within the HPG axis.

### 3.11. qPCR Validation

**Figure 13:**
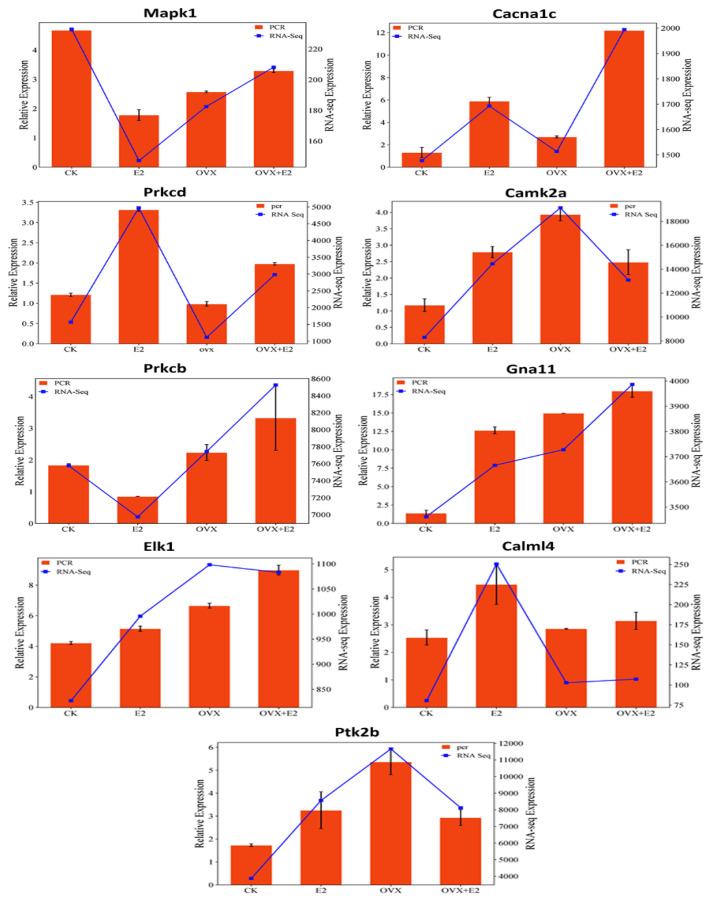
The relative gene expression levels, measured by qPCR and RNA sequencing, for several genes across four mouse groups: control (CK), estradiol-treated (E2), ovariectomized (OVX), and ovariectomized with estradiol (OVX+E2). Each bar graph represents a gene, with orange bars indicating qPCR results and blue lines RNA-seq data. The expression patterns suggest that ovariectomy alters gene expression, which is partially restored with estradiol treatment.

To validate the gene expression patterns identified by RNA sequencing, nine core genes were selected for further analysis using RT-qPCR (Figure 13). These genes—*Mapk12*, *Gna11*, *Cacna1c*, *Elk1*, *Prkcb*, *Calml4*, *Camk2a*, and *Ptk2cb*—were chosen based on their elevated expression in both OVX and E2-treated mice, as well as their key roles in the GnRH signaling pathway.

RT-qPCR analysis confirmed the gene expression trends observed in RNA-seq data. For example, *Prkcd*, *Prkcb*, and *Calml4* were upregulated in E2-treated mice, consistent with RNA-seq results. Similarly, *Elk1* was downregulated in the OVX group compared to OVX+E2, mirroring RNA-seq findings. These results validate the reliability and consistency of the transcriptome data, supporting its suitability for further functional investigations.

### 3.12. Correlation Analysis

**Figure 14:**
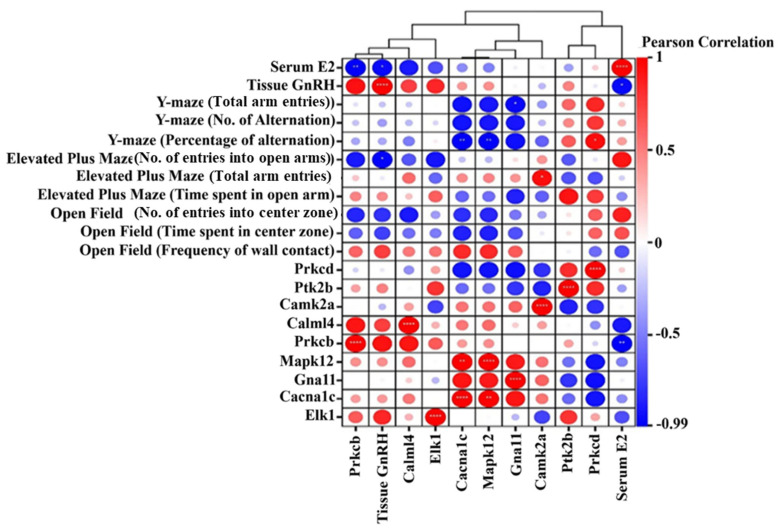
A Pearson correlation matrix showing the relationships between behavioral tests, serum and tissue hormone levels, and gene expression for several genes in an experimental study. Color-coded circles indicate the strength and direction of correlations, with red for positive and blue for negative. The analysis includes data from Y-maze, elevated plus maze, and open field tests, as well as hormone levels (serum E2 and tissue GnRH) and gene expression levels (*Prkcd*, *Ptk2b*, *Camk2a*, *Calml4*, *Prkcb*, *Mapk12*, *Gna11*, and *Cacna1c*). The matrix helps identify potential links between behavior, hormones, and gene expression. * *p* < 0.05; ** *p* < 0.01; **** *p* < 0.0001.

Pearson correlation conducted testing to assess the statistical correlations (*p* < 0.05) between mRNA expression levels and serum hormone levels as well as GnRH amounts and behavioral reactions. The levels of *prkcd* expression in hypothalamic tissue declined and correlated positively with Y-maze measures being behavioral flexibility and exploration (r = 0.855). The relationship between *Prkcb* expression and serum E2 levels showed an exceptionally negative correlation value of r = −0.995, suggesting its role in hormonal modulation. The Y-maze (alternation) shows a significant negative relationship (r = −0.394) with these data points, possibly suggesting memory or cognitive flexibility roles for the molecule. The reduction of Gna11 expression in mouse hypothalamus tissue coupled with a strong negative correlation (r = −0.964) to total Y-maze (arm entries) suggests that Gna11 restricts exploratory behavior by functioning in signal transduction pathways. Exposure to E2 in mouse hypothalamus tissue led to decreased Mapk12 expression while keeping an inverse relationship with the E2 serum concentrations. Serum E2 shows a major negative correlation (R = −0.961, *p* < 0.05) with hypothalamic GnRH expression, which demonstrates that elevated estrogen levels lead to decreased GnRH protein concentration in the hypothalamus region (Figure 14).

## 4. Discussion

Ovariectomized animals are commonly used as models to mimic menopause. In this study, the ovariectomized mice showed no estrous cycle, as confirmed by vaginal exfoliated cell analysis. The success of the ovariectomy procedure was demonstrated by the reduced serum estradiol levels in the OVX mice, confirming the use of these animals as a menopause model [32].

Energy balance directly influences reproductive function across various organisms. Estrogen, a key sex steroid hormone, regulates food intake, energy metabolism, and body weight in mice and other vertebrates, including mammals and humans [33]. In this study, ovariectomy (OVX) led to increased food intake and body weight, consistent with the effects of estrogen deficiency. Estradiol replacement effectively reversed these changes, highlighting estrogen’s role in preventing hyperphagia and excessive weight gain in females. However, some studies report conflicting results, showing decreased food intake but increased body weight following OVX [34].

Our findings demonstrate that E2 treatment in an OVX context significantly influenced exploratory behavior. This finding align with previous research showing that post-training estradiol administration enhances both spatial and object memory consolidation in OVX mice [35], and improves working memory performance in subjects receiving E2 treatment [36]. Our results align with a prior study [37], which demonstrated that elevated or sustained estradiol levels may impair memory through potential dysregulation of estrogen receptor signaling or hippocampal overactivation. Such divergent cognitive effects may arise from critical variations in treatment parameters, including dosage, administration method, and timing [38]. Long-term estradiol exposure following ovariectomy, combined with the absence of natural menstrual cycles, appears to disrupt normal estrogen signal transduction in brain regions regulating working memory—specifically the medial prefrontal cortex and hippocampus. The “critical window hypothesis” further underscores the importance of E2 administration timing after OVX: estradiol provides neuroprotective benefits during a specific post-surgical period, which diminish when treatment initiation is delayed [39]. Collectively, these results highlight the dual hormonal mechanisms through which estradiol influences cognitive function, emphasizing the need for systematic investigation into optimal timing and dosage parameters for hormone replacement therapies.

Our findings also demonstrate that E2 treatment significantly influenced exploratory behavior, as assessed by the open field test. E2-treated mice (E2 and OVX+E2 groups) exhibited increased exploratory activity, with more central zone entries and reduced thigmotaxis, indicating anxiolytic effects. These results align with previous research showing that estrogen therapy reduces anxiety-like behaviors in OVX animals [40,41]. However, OVX mice displayed lower exploratory activity without significant changes in anxiety-like behavior, suggesting that ovariectomy affects exploration more than anxiety [42]. Memory impairment, anxiety, and depressive-like behaviors were also observed following estrogen deprivation, consistent with prior findings [43]. Interestingly, the OVX+E2 group showed lower Y-maze alternation rates compared to the CK, OVX, and E2 groups, suggesting that estradiol replacement may not fully restore cognitive function. These behavioral changes may be linked to alterations in neurotransmitter levels, neuroinflammation, and signaling pathways such as Nrf2/HO-1 [44]. Notably, early estrogen treatment has been shown to improve working memory in OVX animals, emphasizing the importance of timing in hormone replacement therapy [45].

The elevated plus maze test further supported the anxiolytic effects of estradiol, with CK and E2 mice spending more time in open arms, indicating reduced anxiety-like behavior compared to OVX mice. These results align with previous studies demonstrating that estradiol and estrogen receptor-β modulators reduce anxiety in mice [46]. Additionally, GnRH plays a critical role in reproductive function by regulating gonadotropin release from the pituitary gland [47]. Our study found that OVX mice had significantly higher hypothalamic GnRH levels than CK, E2, and OVX+E2 groups, likely reflecting the hypothalamus’s compensatory response to ovarian estrogen loss. Estradiol therapy restored GnRH levels to those observed in control mice, reinforcing estrogen’s well-established negative feedback on GnRH release and regulation of the hypothalamic–pituitary–gonadal (HPG) axis [48].

High-throughput RNA-seq was used to assess hypothalamic gene expression variations associated with reproduction [49]. This approach identified DEGs across six comparisons among four treatment groups in the present study. These results indicate that estradiol therapy profoundly alters hypothalamic gene expression, predominantly through upregulation. Previous studies have shown that continuous estradiol treatment in OVX mice increases estrogen response element (ERE)-dependent gene expression in the hippocampus and cortex but not in the hypothalamus [50], suggesting that estradiol’s transcriptional effects are brain-region specific and influenced by treatment duration. Moreover, estrogen has been shown to modulate gene expression in both the hypothalamus and white adipose tissue, with more pronounced effects in adipose tissue, indicating tissue-specific responses [51].

Bioinformatics analyses, including GO and KEGG pathway enrichment, were conducted to explore the biological significance of the identified DEGs [52]. Hypothalamic DEGs were significantly enriched in GO terms related to cognition, behavioral regulation, reproductive development, placenta formation, and synaptic function, underscoring their relevance to female reproductive processes. Additionally, 17β-estradiol modulates hypothalamic neuronal excitability, impacting reproduction, energy balance, and circadian rhythms [53]. Similarly, estradiol-dependent gene expression analysis in the amygdala of OVX rats revealed downregulation of genes involved in estrogen signaling and synaptic pathways, further supporting estrogen’s role in neural regulation [54].

KEGG analysis showed that DEGs in the hypothalamus of CK, E2, OVX, and OVX+E2 mice were primarily enriched in the calcium signaling pathway, neuroactive ligand–receptor interactions, MAPK signaling, oxytocin signaling, GnRH signaling, and GnRH secretion. Notably, 70 DEGs were identified in the GnRH signaling pathway across the six comparisons. The KNDy neuron model describes kisspeptin, neurokinin B (NKB), and dynorphin as key regulators of GnRH pulse generation [55]. Estrogen modulates KNDy neuron activity through both positive and negative feedback, thereby controlling GnRH secretion and reproductive function [56].

However, this study did not establish direct connections between *Elk1*, *Prkcb*, and *Camk2a* expression changes and KNDy-mediated GnRH regulation. Further research is needed to determine whether estradiol directly modulates KNDy neuron excitability and how these changes influence hypothalamic GnRH secretion. Additionally, decreased *Mapk12* mRNA levels in OVX mice suggest that estrogen loss disrupts MAPK-controlled GnRH regulatory mechanisms. However, it remains unclear whether exogenous E2 can rescue this signaling pathway. A comparative analysis of MAPK signaling dynamics between this study and previous research on OVX and E2-treated models could provide deeper insights into estrogen’s role in hypothalamic regulation.

The activation of *E2* and *Camk2a* in GnRH neurons alters neuronal excitability and synaptic plasticity [57]. In this study, *E2* treatment elevated *Camk2a* expression, potentially enhancing calcium signaling via activated *Camk2a* and increasing pulsatile GnRH release. Previous research has demonstrated that phosphorylation events regulated by *Camk2a* influence GnRH neuron function [58]. Further validation of these findings could be achieved through calcium imaging and *Camk2a* inhibition experiments.

PKC isoforms play a critical role in estrogen receptor activation and subsequent gene transcription [59]. The PKC signaling pathway interacts with estrogen receptors in GnRH neurons to regulate pulsatile GnRH release. This hypothesis could be further supported by experiments using PKC-specific inhibitors and estrogen receptor antagonists. Additionally, estrogen has been shown to regulate both hypothalamic gene expression and neuroplasticity. *E2* replacement in OVX mice significantly increased *Camk2a* and *Elk1* expression, genes associated with synaptic plasticity and neuroprotection [60]. Recent evidence also suggests that *E2* counteracts stress-induced alterations in hypothalamic gene expression, supporting the results of this study [61].

Research on the MAPK pathway has demonstrated that estrogen signaling regulates *Mapk1*, aligning with the inverse gene expression patterns observed between OVX and E2-treated groups [62]. These findings underscore estrogen’s essential role in maintaining hypothalamic function by modulating neural signaling, protecting cells, and enhancing synaptic flexibility. The differential expression of key genes between OVX and OVX+E2 groups further highlights *E2*’s ability to mitigate estrogen deficiency-related outcomes. This knowledge is valuable for advancing neuroendocrine research and could inform the development of hormone therapies for postmenopausal women and individuals with estrogen deficiencies. Future studies should investigate the functional consequences of these molecular changes by integrating behavioral assessments with neurophysiological analyses. Examining how *E2* interacts with neuromodulatory factors presents an opportunity to gain a more comprehensive understanding of estrogen’s influence on hypothalamic function and overall brain health.

A significant negative correlation was observed between Y-maze alternation performance and *Gna11* expression, suggesting that *Gna11* may regulate cognitive flexibility and exploratory behavior. The reduced expression of the *Gna11* G-protein subunit in OVX mice implies decreased synaptic reliance on estrogen. However, this study does not address how *Gna11* downregulation affects cognitive function in estrogen-deficient mice, despite evidence that estrogen regulates G-protein signaling in hippocampal networks [63]. Future research should explore whether estradiol alleviates cognitive deficits via G-protein-coupled receptor (GPCR) signaling pathways.

Additionally, this study found that serum *E2* levels negatively correlated with *Mapk12* expression and a concurrent reduction in hypothalamic MAPK signaling. Given that MAPK pathways regulate stress responses, these changes may contribute to the anxiety-like behaviors observed in OVX mice. However, it remains unclear whether reduced *Mapk12* protein levels directly mediate estrogen’s anxiolytic effects. One possible mechanism is that *E2* downregulates *Mapk12*, thereby reducing MAPK-mediated stress responses and anxiety-like behaviors, particularly in the amygdala. Future studies should examine this potential pathway to clarify the role of estrogen in anxiety regulation.

In the CK_vs_E2 comparison, nine differentially expressed genes (DEGs) related to the GnRH pathway were identified. Notably, *Elk1*, *Ptk2b*, *Camk2a*, *Calml4*, and *Mmp14* were upregulated, while *Egr1*, *Fshb*, *cga*, and *Mapk12* were downregulated, indicating that estradiol modulates GnRH signaling. In the CK_vs_OVX group, 16 genes (including *Elk1*, *Ptk2b*, src, several adenylyl cyclases, and *Camk2a*) were upregulated and three genes (*Mmp2, Nras,* and *Pla2g4a*) were downregulated, suggesting that ovarian hormone loss activates compensatory intracellular pathways. Further comparisons demonstrate that estradiol replacement selectively adjusts the expression of genes—particularly those involved in calcium signaling and related cascades—essential for GnRH neuron function. These results corroborate previous findings on estradiol’s role in regulating GnRH neuronal excitability and intracellular signal transduction [64].

Estradiol exerts both inhibitory and stimulatory effects on GnRH gene expression and release, depending on physiological context [65]. The GnRH signaling pathway plays a crucial role in hormone secretion [66]. Our RNA-seq analysis identified 70 DEGs within the GnRH pathway, with *Mapk12*, *Gna11*, *Cacna1c*, *Elk1*, *Prkcb*, *Calml4*, *Camk2a*, *Ptk2b*, and *Ptk2cb* exhibiting high expression levels. KEGG pathway analysis suggests that the regulatory role of the GnRH system in sex differentiation is conserved across mammals.

A correlation analysis between these nine key genes and phenotypic traits—including serum E2 levels, GnRH expression, and behavioral outcomes—identified *Prkcb* and *Prkcd* as critical DEGs regulating reproductive functions such as meiotic division, egg activation, fertilization, and early embryonic development [67]. *Prkcd* expression in hypothalamic tissue was positively correlated with Y-maze total arm entries and alternations, suggesting a role in behavioral flexibility and exploration. Conversely, *Prkcb* exhibited a strong negative correlation with serum E2 levels, indicating its involvement in hormonal regulation. The positive correlation between *Prkcb* and *Prkcd* suggests their interaction within shared signaling pathways. In the present study, key DEGs including *Elk1*, *Camk2a*, and *Prkcb* were identified as critical regulators of GnRH signaling. As reported in prior rodent pituitary research [68], the MAPK/ERK signaling pathway activates *Elk1* following GnRH stimulation, initiating immediate early gene transcription of c-fos and Egr1. For *Camk2a*, our findings align with evidence that CaMKII-mediated calcium-dependent responses, activated by GnRH, regulate reproductive gene expression [69], whereby *Camk2a* mediates GnRH-evoked calcium signaling cascades. Regarding *Prkcb*, our data provide mechanistic support for its differential expression: this gene is involved in protein kinase C (PKC) signaling pathways that modulate GnRH receptor responsiveness [70]. Two limitations constrain the interpretability of these findings. First, the study utilized a small sample size (*n* = 10 per group), which may limit statistical power and generalizability. Second, the cross-sectional design precluded assessment of dynamic transcriptional changes over time. Research [71] has emphasized the temporal dependency of GnRH signaling, highlighting the need for future longitudinal studies to characterize how gene expression dynamics underpin hormonal regulation of reproductive pathways. These observations underscore the importance of integrating temporal and mechanistic analyses to fully elucidate the molecular networks governing GnRH signaling.

*Camk2a* was significantly enriched in the GnRH signaling pathway. Research indicates that *Camk2a* mobilizes Ca^2^⁺ stores via *ITPR1* and *ITPR2*, promoting LH and FSH expression and secretion [72]. In this study, *Camk2a* expression was elevated in the hypothalamus of E2-treated mice and positively associated with open-arm entries in the elevated plus maze, suggesting a role in neural plasticity and anxiety-related behaviors. Additionally, its negative correlation with Y-maze alternations may indicate an involvement in cognitive flexibility [73].

Similarly, *Gna11*, a key DEG in the GnRH pathway, is known to regulate phosphocalcic homeostasis during lactation. In this study, *Gna11* expression in the hypothalamus was reduced and negatively correlated with Y-maze total arm entries, suggesting that *Gna11* may suppress exploratory behavior, potentially through its role in signal transduction pathways [74]. *Mapk12* is essential for regulating apoptosis and differentiation within the GnRH system, including induced apoptosis in ovarian cells. Here, *Mapk12* expression was downregulated in the hypothalamus and negatively correlated with serum E2 levels, suggesting estrogen-mediated suppression of *Mapk12* expression [75]. Moreover, serum E2 levels showed a significant negative correlation (r = −0.961, *p* < 0.05) with hypothalamic GnRH levels, supporting the established negative feedback loop wherein elevated estrogen levels suppress GnRH expression. In this study, OVX mice exhibited higher E2 concentrations than OVX+E2 mice but also had higher GnRH concentrations, reinforcing the compensatory role of the hypothalamus in response to estrogen depletion [76].

Although our study sheds light on the dynamic molecular changes in hypothalamic tissue during mouse reproduction, several limitations must be acknowledged. First, the relatively small sample size and the lack of complementary molecular validations may limit the reliability and robustness of our findings. Second, it is important to examine the impact of modulating the PKC and MAPK pathways on GnRH expression and associated behavioral responses, as these could play critical roles in the observed effects.

## 5. Conclusions

This study identified eight keys differentially expressed hypothalamic genes, *Prkcd*, *Prkcb*, *Mapk12*, *Gna11*, *Camk2a*, *Cacna1c*, *Elk1*, and *Ptk2b*, that are involved in reproductive signaling pathways. Behavioral analyses demonstrated that estradiol exposure alters anxiety-related responses and exploratory behaviors, suggesting a functional association between hypothalamic gene expression changes and reproductive neural regulation. These findings highlight the role of transcriptional alterations in mediating hormonal influences on behavioral and neuroendocrine mechanisms underlying reproductive physiology.

## Data Availability

The data sets that were generated and/or analyzed in the current study are available from the corresponding author upon reasonable request.

## Supplementary Materials

The following supporting information can be downloaded at: https://www.mdpi.com/article/10.3390/ani15101467/s1, Table S1: Indicate the anxiety index for open field test, data were expressed as the mean ± standard deviation (SE); Table S2: Indicate the anxiety index for Y-Maze, data were expressed as the mean ± standard deviation (SE); Table S3: Indicate the anxiety index for Elevated plus maze, data were expressed as the mean ± standard deviation (SE); Table S4: Summary of quantitative PCR primers of target and reference genes in mice hypothalamus.
