# Supplementary material for: Estradiol Reverses Ovariectomy-Induced Disruption of Hypothalamic Gene Expression and Behavior via Modulation of Gonadotropin Releasing Hormone and Calcium Signaling Pathways
Source: Animals (Basel). 2025 May 19;15(10):1467. doi: 10.3390/ani15101467 (PMC12108420; doi:10.3390/ani15101467)
Supplement: Supplementary file 1 [file animals-15-01467-s001.zip › animals-3598867-supplementary.pdf]

**Table S1:** Indicate the anxiety index for open field test, data were expressed as the mean ± standard deviation (SE)

| Measure | Days | Condition | | | |
|---|---|---|---|---|---|
| | | CK | E2 | OVX | OVX+E2 |
| Open field (No. of Entries into center zone) | 1-4 | 8.28±4.15 | 8.07±6.19 | 4.71±3.45 | 4.78±2.75 |
| Open field (No. of Entries into center zone) | 5-8 | 7.42±3.03 | 6.71±2.05 | 4.50±2.17 | 6.07±3.14 |
| Open field (No. of Entries into center zone) | 9-13 | 6.71±3.22 | 6.71±2.05 | 4.50±2.17 | 6.28±2.46 |
| Open field (Time spend in center zone) | 1-4 | 8.72±7.63441 | 7.28±4.39 | 4.01±3.61 | 6.10±4.62 |
| Open field (Time spend in center zone) | 5-8 | 9.70±6.97 | 9.02±6.61 | 3.76±3.58 | 6.20±3.42 |
| Open field (Time spend in center zone) | 9-13 | 13.46 ±12.41 | 7.98±4.24 | 5.28±4.15 | 8.43±5.02 |
| Open field (Frequency of wall contact) | 1-4 | 6.57±2.47 | 7.42±4.36 | 9.35±2.37 | 8.00±3.28 |
| Open field (Frequency of wall contact) | 5-8 | 6.57±1.60 | 8.35±5.07 | 9.71±3.02 | 8.50±3.71 |
| Open field (Frequency of wall contact) | 9-13 | 6.78±2.93 | 7.71±2.64 | 9.21±2.51 | 8.00±3.32 |

**Table S2:** Indicate the anxiety index for Y-Maze, data were expressed as the mean ± standard deviation (SE)

| Measure | Days | Condition | | | |
|---|---|---|---|---|---|
| | | CK | E2 | OVX | OVX+E2 |
| Y-Maze (No of Alternation) | 1-4 | 13.28±5.78 | 11.42±5.04 | 9.14±5.47 | 8.57±5.37 |
| Y-Maze (No of Alternation) | 5-8 | 12.57±5.62 | 8.57±5.10 | 7.07±5.79 | 8.50±2.84 |
| Y-Maze (No of Alternation) | 9-13 | 13.28±5.60 | 7.78±3.19 | 7.85±5.48 | 7.71±2.70 |
| Y-Maze (Percentage of Alternation) | 1-4 | 64.76±10.68 | 61.76±8.63 | 57.65±17.66 | 57.73±14.02 |
| Y-Maze (Percentage of Alternation) | 5-8 | 62.77±9.75 | 55.72±11.80 | 48.17±24.30 | 46.03±22.18 |
| Y-Maze (Percentage of Alternation) | 9-13 | 63.63±12.04 | 57.84±14.45 | 52.72±17.54 | 50.52±15.75 |
| Y-Maze (No of Total arm Entries) | 1-4 | 21.14±6.89 | 19.21±5.97 | 15.71±5.26 | 13.85±5.66 |
| Y-Maze (No of Total arm Entries) | 5-8 | 21.42±9.42 | 15.92±6.36 | 15.71±6.78 | 14.85±4.43 |
| Y-Maze (No of Total arm Entries) | 9-13 | 24.42±5.35 | 14.92±3.19 | 15.71±6.68 | 15.07±3.26 |

**Table S3:** Indicate the anxiety index for Elevated plus maze, data were expressed as the mean ± standard deviation (SE)

| Measure | Days | Condition | | | |
|---|---|---|---|---|---|
| | | CK | E2 | OVX | OVX+E2 |
| Elevated plus maze (Time spend in open arm) | 1-4 | 20.05±12.14 | 12.91±14.01 | 17.54±15.94 | 8.43±9.52 |
| Elevated plus maze (Time spend in open arm) | 5-8 | 28.44±21.12 | 13.77±16.35 | 20.54±20.87 | 12.54±10.03 |
| Elevated plus maze (Time spend in open arm) | 9-13 | 27.60±16.92 | 15.57±15.19 | 26.38±22.49 | 8.67±10.80 |
| Elevated plus maze (No of Entries into open arm) | 1-4 | 3.50±2.13 | 2.21±1.84 | 2.30±1.25 | 1.73±1.62 |
| Elevated plus maze (No of Entries into open arm) | 5-8 | 3.21±2.72 | 1.85±1.74 | 3.38±1.55 | 1.60±1.05 |
| Elevated plus maze (No of Entries into open arm) | 9-13 | 3.92±2.12 | 2.35±1.82 | 3.84±1.99 | 2.20±2.04 |
| Elevated plus maze (Total arm Entries) | 1-4 | 9.42±4.92 | 7.42±4.05 | 7.61±2.53 | 6.06±1.70 |
| Elevated plus maze (Total arm Entries) | 5-8 | 9.71±6.77 | 5.57±2.34 | 8.07±2.32 | 7.53±2.47 |
| Elevated plus maze (Total arm Entries) | 9-13 | 9.42±2.95 | 6.35±4.65 | 9.35±2.90 | 6.80±3.42 |

**Table S4:** Summary of quantitative PCR primers of target and reference genes in mice hypothalamus

| Genes | primers | Sequences (5`-3`) |
|---|---|---|
| Mapk12 | F | CCTATGGTGCAGTGTGCTCT |
| | R | GTGCCCATGAATGGCATCAC |
| Gna11 | F | CCCTGGTTCCAGAACTCGTC |
| | R | ATCCCTCTGTGGCCCATCAA |
| Calml4 | F | GGATGGCCAAGTTCCTTTCC |
| | R | GCTCTCCGTTCTTGTCTATCC |
| Elk1 | F | CAGGAATGACAGGCCAAGGT |
| | R | GGTGGGGTTAGGATAACCTGC |
| Cacna1c | F | CCTGGCCATGCAGCACTAT |
| | R | GCTCCCAATGACGATGAGGA |
| Prkcb | F | GTGGCGTATCCCAAGTCCAT |
| | R | GTCTCGCTTGTCTCTAGCTT |
| Ptk2b | F | TCCACCGGCACAATGTCTTC |
| | R | GGTTTCTGTGGGGGCCTGTAG |
| Camk2a | F | CAGCATCCCAGCCCTAGTTC |
| | R | GAGAAGGCTCCCTTTCCCAG |
| Prkcd | F | CTGGGAGAGTCCCTTCCTCA |
| | R | GCTGCATAAAACGTAGCCCG |
| B-Actin | F | CTGTCGAGTCGCGTCCACC |
| | R | ATTCCCACCATCACACCCTGG |
